# A Novel del(20q) in Aggressive Nodal Marginal Zone Lymphoma

**DOI:** 10.1155/2013/784176

**Published:** 2013-02-12

**Authors:** Jason B. Kern, Deiter J. Duff, Jamie L. Odem, Magda Esebua, Lisa R. Smith, Donald Doll, Michael Wang

**Affiliations:** ^1^Department of Pathology and Anatomical Sciences, School of Medicine, University of Missouri, Columbia, MO 65203, USA; ^2^Department of Cytogenetics, Sonora Quest Laboratories, Tempe, AZ 86281, USA; ^3^Division of Hematology and Medical Oncology, Ellis Fischel Cancer Center, School of Medicine, University of Missouri, Columbia, MO 65203, USA

## Abstract

This is a case report of a previously undescribed 20q chromosomal deletion (del(20q)) in marginal zone lymphoma (MZL). A 54-year-old Caucasian male presented with an enlarging neck mass and multiple violaceous skin nodules over his chest. Biopsy of the neck mass and cervical lymph nodes revealed MZL. Cytogenetic evaluation of both lymph node and bone marrow tissue revealed del(20q). This was an unexpected finding, as del(20q) is associated with myelodysplastic syndromes and myeloproliferative neoplasms and rarely seen in diffuse large B-cell lymphoma, follicular lymphoma, and T-cell lymphoma, but has not previously been described in MZL. We describe the case presentation and histologic findings and discuss the significance of this novel finding.

## 1. Introduction

20q chromosomal deletion (del(20q)) is a well-described cytogenetic abnormality in myeloid neoplasms such as myelodysplastic syndrome (MDS), acute myeloid leukemia (AML), and the myeloproliferative neoplasms (MPN) [1]. Del(20q) is rare in lymphomas, but has been detected in cases of diffuse large B-cell lymphoma, follicular lymphoma, and T-cell lymphoma [1]. A literature search and the National Cancer Institute's Recurrent Chromosome Aberrations in Cancer Database failed to reveal any reported cases of del(20q) in marginal zone lymphoma (MZL) [2]. Here we present the first reported case of MZL with del(20q), detected by FISH in lymph node tissue and by karyotyping in involved bone marrow tissue. 

## 2. Case Presentation

The patient is a 54-year-old Caucasian man with a slowly enlarging neck mass, accompanied by multiple violaceous skin nodules over his chest and neck and B symptoms. Physical exam revealed an 11 × 8 cm tender, erythematous right neck mass as well as multiple violaceous, mobile skin nodules on the neck and chest. Splenomegaly was not present. Also of note, the patient is hepatitis C virus positive, which is associated with MZL [3]. A CT of the neck revealed a large, poorly defined right neck mass measuring up to 9 cm as well as many enlarged cervical lymph nodes. An open biopsy was performed and a right posterior cervical lymph node and tissue from the deep neck mass were sent for histologic evaluation.

**Figure 1:**
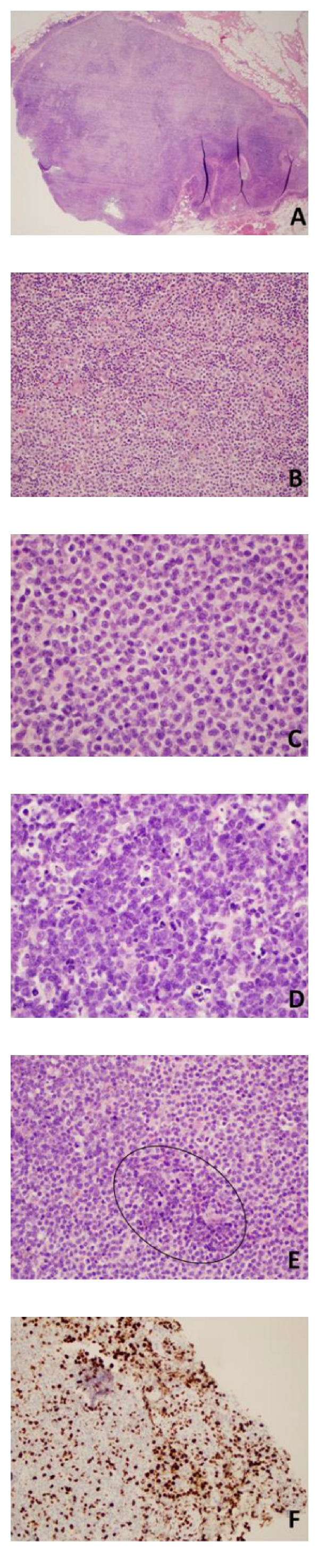
Histological features of lymph node. (A and B) Right posterior cervical lymph node, (A) H&E, 2x. The node is nearly completely effaced with minimal residual follicles. (B) H&E, 20x. Sheets of monocytoid cells in marginal zone. (C–F) Right cervical neck mass. (C) H&E, 60x. Sheets of monocytoid cells similar to those seen in the cervical lymph node. (D) H&E, 60x. Area of larger cells with focal necrosis. (E) H&E, 40x. Monocytoid cells and groups of larger cells (circle) with hyperchromasia. (F) MIB1 immunostain, 20x. Transformation areas with large cells show MIB-1 labeling index as high as 70%.

Microscopic examination showed almost complete effacement of the node (Figure 1(A)). Follicles were replaced by atypical monocytoid cells (Figures 1(B) and 1(C)), which stained positive for PAX-5, CD19, and CD20, but were negative for CD5 and cyclin D1. The effaced area had a MIB-1 labeling index of 10%. BCL-6 and CD23 were diffusely positive in remnant germinal centers which were replaced by atypical monocytoid cells, and these areas corresponded with a negative CD10. These morphologic and immunohistochemical (IHC) findings are characteristic of a nodal MZL [3, 4].The tissue from the deep neck mass revealed similar histology but an area with larger, more hyperchromatic cells (Figures 1(D) and 1(E)) and a higher mitotic rate, with a MIB-1 labeling index of up to 70% (Figure 1(F)). The neoplastic lymphocytes invaded beyond the capsule into the surrounding muscle, consistent with an aggressive morphology [4]. Flow cytometry of the neck mass revealed a monoclonal B-cell population expressing kappa immunoglobulin light chains, CD19 and CD20.

**Figure 2:**
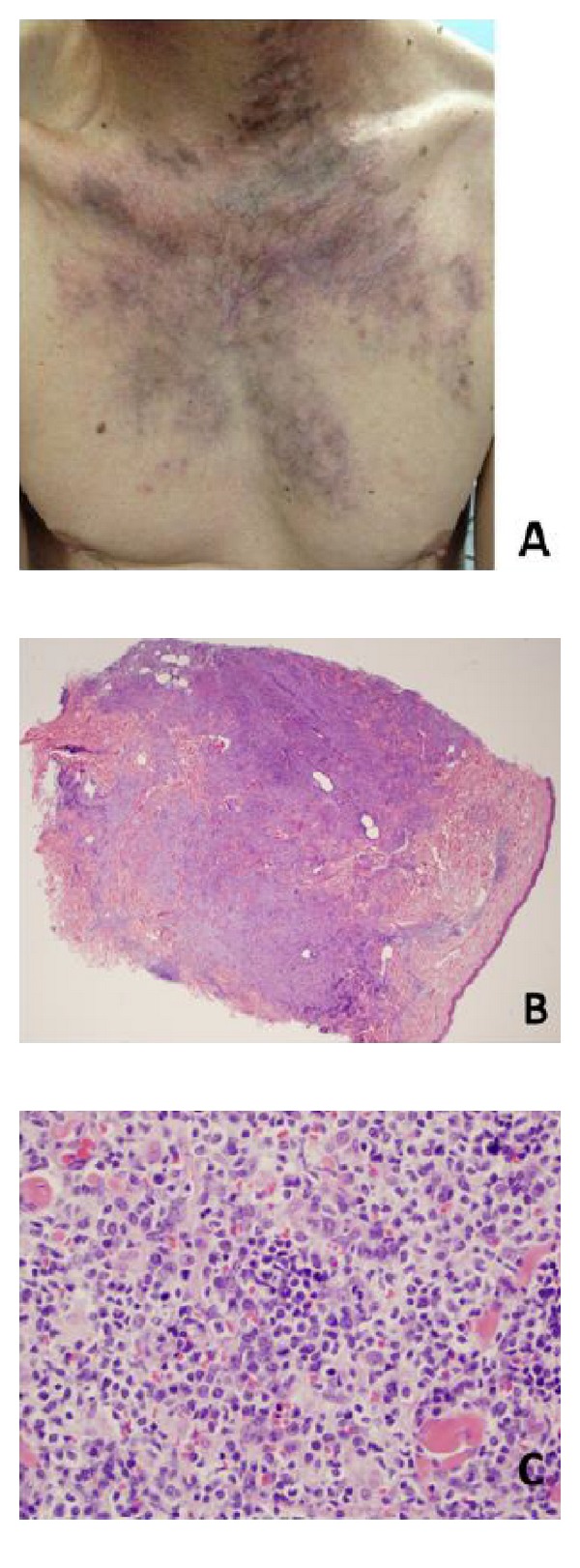
Chest skin lesion. (A) Clinical photo of violaceous skin lesion on the patient's chest. (B) Punch biopsy, H&E, 2x. MZL cells invading the dermis. (C) Punch biopsy, H&E, 40x. Small lymphoid cells consistent with MZL are observed here, but also the larger, more hyperchromatic cell population with many mitotic figures, similar to findings in the transformation areas of neck mass.

Punch biopsy of one of the skin nodules (Figure 2(A)) showed diffuse involvement of the dermis (Figure 2(B)). The histology showed mostly small monocytoid B cells (Figure 2(C)), again with a classic MZL immunophenotype: BCL6 and CD23 positive in remnant follicles replaced by atypical CD20 positive monocytoid B cells.

**Figure 3:**
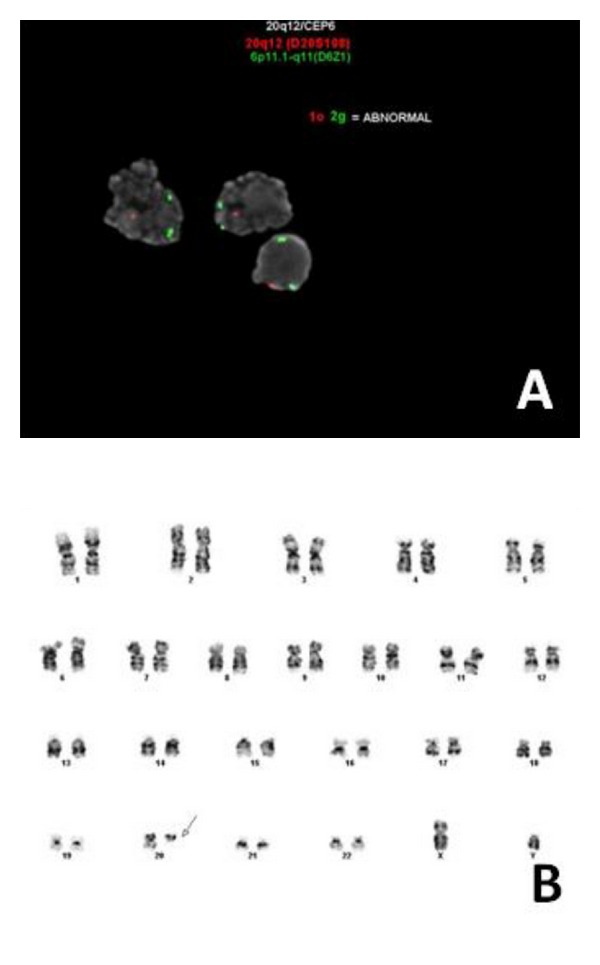
FISH and cytogenetics analysis. (A) FISH performed on a touch preparation of representative lymph node tissue shows only one positively tagged 20q12 (red signal) per cell. Control tag 6p11 (green) shows dual positivity. FISH is diagnostic for del(20q12). (FISH probes from Abbott Molecular, Inc.) (B) Karyotype of bone marrow aspirate. Cytogenetics performed on bone marrow tissue reveals deletion of 20q11.2 (arrow).

Both lymph node and bone marrow tissues were sent for cytogenetic evaluation. FISH on representative lymph node tissue revealed del(20q), near 20q12 (Figure 3(A)). There was not morphologic evidence of MZL in the bone marrow nor any myeloid neoplasm or other 20q deletion-associated disease identified. However, a karyotype of the bone marrow aspirate also revealed a del(20q), near 20q11.2 (Figure 3(B)). This may represent an enrichment of MZL cells during cell culture and molecular evidence of MZL involvement in the bone marrow. The patient was treated with bendamustine plus rituximab and responded well with a complete remission.

## 3. Discussion

Nodal MZL is one of three subtypes of MZL and its incidence is relatively rare, comprising only 1.5% of all lymphomas [3], characterized by its monocytoid tumor cells in an expanded marginal zone of lymph node. Disease onset is usually around 60 years of age, typically involves cervical and/or inguinal lymph nodes, and is often of advanced stage at diagnosis. Immunophenotype is CD19+, CD20+, PAX5+, BCL2+, CD5−, CD23−, and cyclin D1−. Genetic abnormalities are common, including trisomy 3, trisomy 7, trisomy 12, trisomy 18, and del6q [3]. Other less common abnormalities include losses and/or gain in chromosomes 1, 4, 6, 7, 8, 13, and 21, but not 20 [5].

The finding of a del(20q) in both the lymph node tissue and bone marrow samples was an unexpected finding in this case; a diagnosis of nodal MZL could confidently be made by lymph node histological features, IHC and flow cytometric immunophenotyping. The literature has not previously reported del(20q) in MZL. This finding may represent a specific cytogenetic abnormality in this nodal MZL with an aggressive morphology and high-stage disease. In conclusion, this case of nodal MZL with 20q deletion is a novel finding which can expand the breadth of knowledge of chromosomal abnormalities in MZL.
